# Clinical consequences of gestational diabetes mellitus and maternal obesity as defined by asian BMI thresholds in Viet Nam: a prospective, hospital-based, cohort study

**DOI:** 10.1186/s12884-022-04533-1

**Published:** 2022-03-09

**Authors:** Serena Yue, Vu Thai Kim Thi, Le Phuong Dung, Bui Thi Hong Nhu, Evelyne Kestelyn, Dang Trong Thuan, Le Quang Thanh, Jane E Hirst

**Affiliations:** 1 Nuffield Department of Women’s & Reproductive Health, University of Oxford, Oxford, UK; 2 The Oxford University Clinical Research Unit (OUCRU), Ho Chi Minh City, Viet Nam; 3 Tu Du Hospital, Ho Chi Minh City, Viet Nam; 4 The George Institute for Global Health, London, UK; 5 Nuffield Department of Women’s & Reproductive Health, John Radcliffe Hospital, OX3 9DU Oxford, UK

**Keywords:** Asian BMI threshold, Gestational diabetes mellitus, Large for gestational age, Maternal obesity, Perinatal outcomes, Preterm birth, Primary C-section, South East Asian

## Abstract

**Background:**

Gestational Diabetes Mellitus (GDM) is common in South East Asia, occurring at relatively lean Body Mass Index (BMI). Outside pregnancy, cardiometabolic risks increase at lower BMI in Asian populations, justifying Asian-specific thresholds for overweight and obesity. We aimed to explore the effects of GDM and obesity on perinatal outcomes using a WHO expert consultation-recommended Asian-specific definition of obesity.

**Methods:**

This is a secondary analysis of a prospective, hospital-based, cohort study in Ho Chi Minh City. Participants were recruited from antenatal clinics between 19^+ 0^-22^+ 6^ weeks gestation and followed until delivery. GDM screening occurred between 24 and 28 weeks using WHO criteria. Obesity was defined as BMI ≥ 27.5 kg/m^2^, based on weight and height at recruitment. We assessed associations between GDM (singly, and in combination with obesity) and perinatal outcomes. Participants were categorised into four groups: no GDM/non-obese (reference group), GDM/non-obese, no GDM/obese and GDM/obese. Outcomes included primary caesarean section, hypertensive disorders of pregnancy (HDP), large-for-gestational-age (LGA), birth weight, preterm birth, and composite adverse neonatal outcome. Logistic and linear regressions were performed with adjustment for differences in baseline characteristics.

**Results:**

Among 4,970 participants, 908 (18%) developed GDM. Compared to women without GDM, GDM increased risks for preterm birth (OR: 1.40, 95% CI: 1.09–1.78), higher birthweight (birthweight z-score 0.16 versus 0.09, *p* = 0.027), and LGA (OR 1.14, 0.89–1.46). GDM without obesity was associated with an increased risk of preterm birth (OR 1.35, 1.04–1.74). Obese women without GDM were more likely to deliver by caesarean section and have an LGA baby (1.80, 1.33–2.44 and 2.75, 1.88–4.03). The highest risks were observed amongst women with both GDM and obesity: caesarean Sect. (2.43, 1.49–3.96), LGA (3.36, 1.94–5.80) and preterm birth (2.42, 1.32–4.44).

**Conclusions:**

GDM was associated with an increased risk of preterm birth and larger newborn size. Using an Asian-specific definition of obesity, we demonstrate obese women with GDM are at the highest risk of adverse outcomes. Using a BMI threshold in pregnancy of 27.5 kg/m^2^ (between 19 and 22 weeks gestation) for Asian women can identify women who will benefit from intensified diabetes, nutritional, and obstetric care. This has relevance for obstetric service delivery within Asia, and other health systems providing pregnancy care for Asian expatriate women.

## Synopsis

Gestational Diabetes Mellitus is associated with adverse pregnancy outcomes (preterm birth, larger newborn size at birth, and caesarean section). These outcomes are exacerbated by obesity (as defined by Asian BMI thresholds) in a Vietnamese population.

## Background

Gestational diabetes mellitus (GDM) is the new onset of glucose intolerance during pregnancy and affects around 15% of pregnancies worldwide, equivalent to 21 million births annually [1, 2]. GDM is usually diagnosed about 24–28 weeks gestation, with the diagnostic gold standard being the oral glucose tolerance test (OGTT). GDM can lead to complications during pregnancy and birth [3], including increased need for delivery by Caesarean section (C-section), hypertensive disorders of pregnancy (HDP) and large-for-gestational-age (LGA) babies. Associations with other adverse neonatal outcomes have been less consistently demonstrated [4–6].

For decades there has been controversy over glycaemic thresholds used to define GDM. The WHO/IADPSG criteria for diagnosis of GDM was released following the Hyperglycaemia and Adverse Pregnancy Outcomes (HAPO) Study [7], and is based on the risk of adverse perinatal outcomes [8].

To date, most research on the consequences of GDM has been conducted in western populations. GDM is common in South-East Asia (SEA), affecting up to 18% of pregnant women [9], however adverse outcomes have been less consistently observed. For example, two studies reported no association between GDM and C-Sects. [10, 11], and only weak associations with pregnancy-induced hypertension and LGA babies [11, 12]. These differences could be due to study design, genetic, metabolic, behavioural, or therapeutic differences. However, it is notable that women in SEA with GDM usually have much lower BMIs than their western counterparts, upon whom much of our understanding of GDM is based.

During pregnancy, obesity and GDM are found to act synergistically to increase the risk of adverse perinatal outcomes in Western populations [13]. However, as populations across Asia experience economic and nutritional transition, the proportion of people living with obesity is increasing. The effects of this on population health are concerning, particularly as cardiometabolic complications occur in Asian populations at relatively lower BMI than people with European (white) ancestry [14]. During pregnancy, the adverse consequences of GDM associated with obesity could follow this pattern, with increased risk observed at BMI levels below the WHO definition of obesity (30 kg/m^2^).

Therefore, we aim to examine the association between GDM and adverse pregnancy outcomes and explore how obesity, (using a definition of BMI ≥ 27.5 kg/m^2^ as recommended by a WHO expert consultation for Asian populations) modulates these risks in an Asian (Vietnamese) urban population.

## Methods

The current study is a secondary analysis from the Viet Nam Preterm Birth Biomarkers (PBB) Project. The PBB was a prospective cohort study conducted between October 2016 to October 2018 in Tu Du Hospital in Ho Chi Minh City, the largest maternity hospital in Viet Nam. Viet Nam is a rapidly developing country with a population of 90 million, GDP per capita of USD 10,755 (Purchasing Power Parity) in 2020, and a maternal mortality ratio of 211 per 100,000 live births in 2017 [15].

From October 2016 until May 2018, women between 19^+ 0^ to 22^+ 6^ weeks gestation were recruited from the hospital’s antenatal outpatient service. Inclusion criteria were maternal age > 18 years, singleton pregnancy, intention to deliver at Tu Du Hospital, ability to give written informed consent, certainty in pregnancy dating from ultrasound scan < 14 weeks gestation, and no signs or symptoms of a threatened miscarriage. Participants were followed up through pregnancy, labour and birth, until the last mother-newborn pair was discharged (last participant October 2018). For this secondary analysis, further exclusions were applied to women who delivered at < 24 weeks gestation (as this preceded the gestation of GDM diagnosis).

The primary exposure for this study was GDM, studied both singly and in combination with obesity. We felt this two-step approach in studying these risk factors (GDM and obesity) is appropriate as GDM has a high prevalence in Asian populations so it is important to look at its associated adverse outcomes. However, it is also important to isolate any independent effects of BMI, particularly given the metabolic overlaps between GDM and obesity.

GDM screening is universal in this hospital as part of routine clinical care. GDM was defined using the IADPSG/WHO criteria (fasting ≥ 5.1 mmol/L; 1-hour ≥ 10.0 mmol/L; 2-hour ≥ 8.5mmol/L). The diagnosis was by 75-gram OGTT between 24 and 28 weeks gestation. BMI was measured from height and weight measurements collected at recruitment (19^+ 0^ to 22^+ 6^ weeks gestation). Height was measured using an Adult Stadiometer (Seca 242 Digital Display), and weight measured using an Adult Scale (Seca 877 Portable Digital Scale) at recruitment. The definition of BMI in Asian populations is controversial [14]. In 2004 a WHO expert group published recommendations for BMI thresholds for overweight and obesity for Asian populations. This concluded that despite differences in the BMI where cardiometabolic risk was increased, as there were no consistent thresholds, the universal definition of obesity should be used. However within this document, Asian-specific trigger points for public health action were given (underweight < 18.5 kg/m^2^, normal 18.5–23 kg/m^2^, overweight 23-27.5 kg/m^2^, obese ≥ 27.5 kg/m^2^) [14]. Given the possibility that adverse consequences associated with obesity could be occurring at lower BMI in this population, we used the Asian trigger point definitions of BMI category for this study.

To study the associations of GDM and obesity in combination, participants were divided into four mutually exclusive groups: (1) no GDM, no obesity; (2) GDM, no obesity; (3) no GDM, obesity; and (4) GDM, obesity. Multivariable logistic regression was used to study the association of GDM and obesity on the three most common adverse outcomes (any C-section, LGA, and preterm birth). Women with no GDM and no obesity were used as the reference group.

We excluded women with pre-existing diabetes (either Type 1 or Type 2 Diabetes). Potential confounding exposures included: maternal age, gestational weight gain from either self-reported pre-pregnancy or weight measured early in conception until recruitment, socioeconomic status (using occupation as a proxy measure), parity, history of hypertension, and history of preterm birth.

Maternal and newborn outcomes associated with GDM were selected based on previously observed associations ranked by consensus [16]. Outcomes assessed were: primary C-section, any C-section (including women who delivered by C-section in earlier pregnancies); HDP including either gestational hypertension (systolic BP ≥ 140 mmHg, diastolic BP ≥ 90 mmHg), pre-eclampsia, eclampsia, or HELLP (haemolysis, elevated liver enzymes, and low platelets); and newborn size at birth using birthweight, birth weight z-score, LGA and SGA (small-for-gestational-age), defined respectively as > 90th and < 10th percentile according to the INTERGROWTH-21st Newborn Size Standards for gestational age at birth and newborn sex [17]. Macrosomia was defined as birthweight > 4000 g. Preterm birth was defined as delivery at < 37 weeks gestation. We used a composite indicator of a severe neonatal adverse outcome as per the ACHOIS Study [18], comprising: stillbirth, neonatal death before discharge, neonatal intensive care unit (NICU) admission, fractured clavicle, transient tachypnoea, or respiratory distress syndrome.

All data were recorded by trained research midwives on paper case record forms and electronically transcribed. Data were collected at two time points: recruitment, and after birth before discharge from the hospital. Trained research midwives interviewed the women at each visit, with medical details obtained from medical records. Women who delivered in other hospitals were interviewed by telephone for basic delivery information. Data were transcribed and stored in a secure database (CliRes).

The sample size was based on the PBB study’s primary objectives. Post hoc, we estimated that for 900 women with GDM compared to 4,000 controls, for outcomes at 5% prevalence in the control group, a risk difference of 2% could be demonstrated, with 80% power and statistical significance set at a two-tailed *p*-value of < 0.05.

Participants loss-to-follow-up were excluded, yielding a complete case analysis. As loss-to follow-up rates were extremely low (0.2%), no imputation was performed for missing values. For weight-related outcomes (LGA and weight z-score), implausible outliers for weight z-score with > or < than three standard deviations (SD) from the mean were dropped. Participant recruitment and subsequent exclusion are presented in a flow diagram.

Univariate analyses used Student T-test for normally distributed variables, Mann-Whitney *U-*test for skewed continuous variables, chi-squared test of homogeneity for categorical variables, and chi-squared test for linear trend for ordinal variables. A 5% two-sided significance level was used for all *p*-values. Box-and-whisker plots were constructed for newborn weight (grams) and weight z-score. Multivariable logistic regression reported Odds Ratios (OR), and multivariable linear regression reported beta coefficients with 95% confidence intervals (CI). Continuous variables were checked for the assumption of log-linear association by testing for departure from linearity using the Likelihood Ratio Test. Covariates that showed a significant association with both exposure and outcome and were not on the causal pathway were assessed for confounding using the chi-squared values from the Likelihood Ratio Test of each serial adjustment for covariates with and without the primary exposure. This helped to gauge how much statistical information the primary exposure contributed to model fit. The decision for adjusting for relevant confounders were informed by both statistical criteria and biological plausibility based on our clinical knowledge and understanding of the literature, Variance Inflation Factors were used to check for multicollinearity, and model diagnostics for linear regression included homoscedasticity, assumption of linearity, and normality of residuals.

To assess the potential of unmeasured confounders explaining away an observed association, the E-Value was computed for outcomes with evidence of significant association [19].

All analyses and plots were performed using STATA 16.1 (StataCorp LLC, Texas, USA).

## Results

**Fig. 1:**
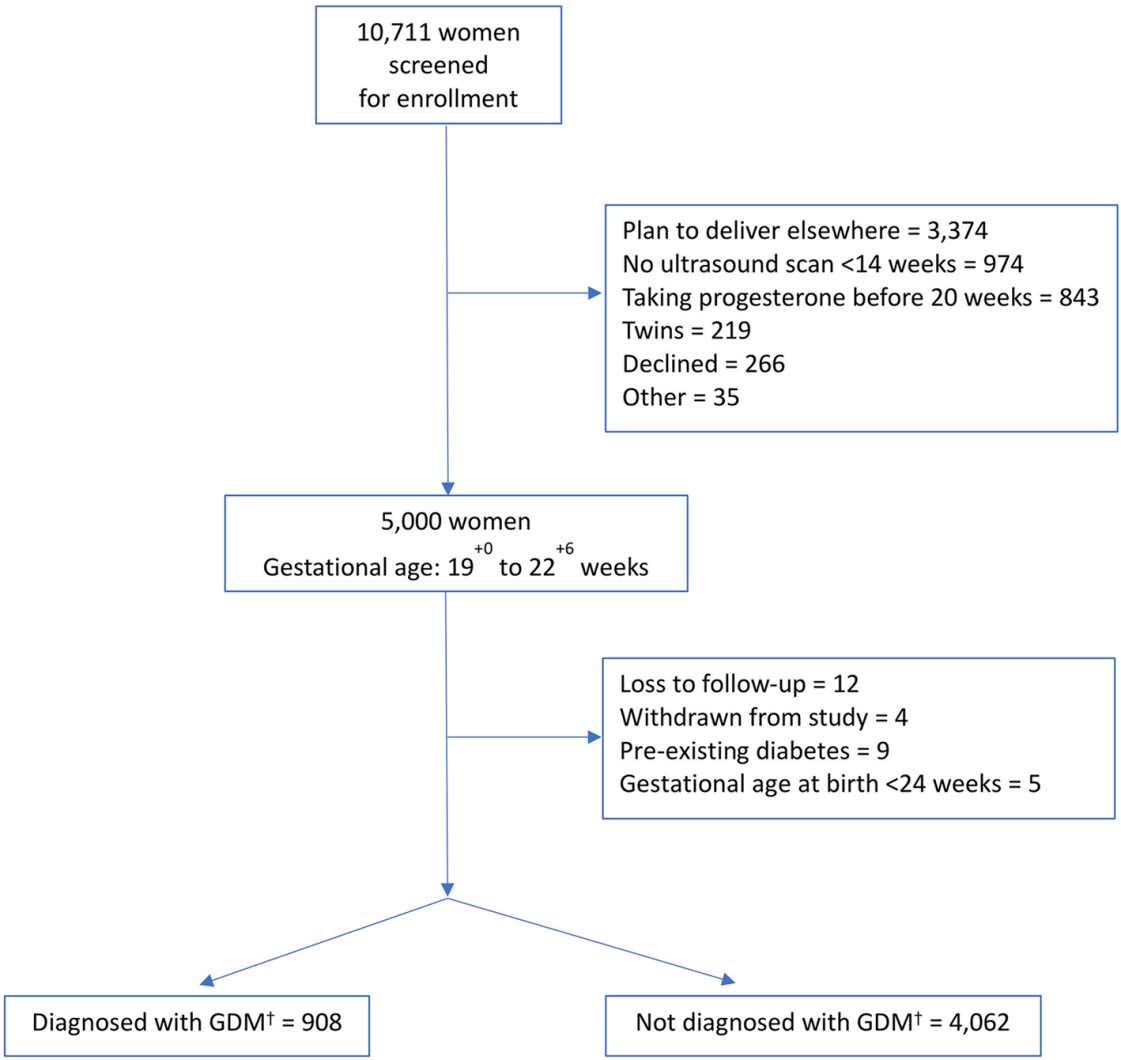
Flow Diagram of Study Participants. † Gestational Diabetes Mellitus; flow diagram showing participant recruitment from enrolment to corresponding numbers of women who were and were not diagnosed with gestational diabetes. Reasons and number of exclusions are stated accordingly

From 10,711 pregnant women screened, 5,000 met the PBB entry criteria and gave consent for participation. After enrolment, 12 women were loss-to-follow-up, and four withdrew. In addition, we excluded nine women with pre-existing diabetes and five who delivered < 24 weeks gestation, leaving 4,970 women and babies for analysis, Fig. 1.

### Cohort characteristics

There were 908 women (18%) diagnosed with GDM, of whom 19 (2%) were treated with insulin.

The mean BMI was 22.5 kg/m^2^ in women without GDM, compared to 23.5 kg/m^2^ in those with GDM (*p* < 0.001). Among the 267 (5.4%) women classified as obese as per the Asian threshold (27.5 kg/m^2^); 189 (4.7%) did not have GDM, while 78 (8.6%) had GDM. Among the 1,895 women classified as overweight as per the Asian threshold (23–27.5 kg/m^2^); 1,466 (36%) did not have GDM, while 429 (47%) had GDM. Among 4,970 women, 78% were neither diagnosed with GDM nor obese, 17% had GDM but were not obese, 3.8% did not have GDM but were obese, and 1.6% had both GDM and obesity. Compared to women without GDM and who were not obese, women with GDM and were obese were significantly older (*p* < 0.001), Table 1. A similar trend was also seen in gestational weight gain; women with GDM and obesity gained on average 2 kg more weight by recruitment (around 21 weeks gestation), (*p* < 0.001). In order to account for the fact that women were recruited at slightly different gestational ages (19–22 weeks), we also looked at the average weight gain per week throughout gestation, and this difference remained significant; obese and GDM women gained more weight per week than women without GDM and not obese (*p* < 0.001). In addition, obese women with GDM were more likely to have had a history of hypertension compared to women who had neither GDM nor obesity (3.9% versus 0.2%, *p* < 0.001). Women with both GDM and obesity were also more likely to be multiparous compared to those women with neither GDM nor obesity (60% versus 45%, *p* < 0.001), as well as more likely to have a history of giving birth to a macrosomia baby/ies; 11% versus 2%, *p* < 0.001.

**Table 1:** Maternal Baseline Characteristics by Gestational Diabetes Mellitus and Obesity Status

	Baseline characteristics					
		No GDM, no obesity	GDM, no obesity	No GDM, obesity	GDM, obesity	***P***-value
Total N (%)	4970	3873 (78)	830 (17)	189 (3.8)	78 (1.6)	
Age (years) Mean (SD)		28.5 (4.3)	30.3 (4.5)	30.4 (5.0)	31.4 (5.7)	<0.001
Marital status N (%)						
Married		3863 (99.7)	829 (99.9)	189 (100)	78 (100)	0.973
Divorced, separated, or single		10 (0.3)	1 (0.1)	0 (0)	0 (0)	
Highest education level attained N (%)						<0.001
No school attended/Primary		57 (1.5)	13 (1.6)	9 (4.8)	5 (6.4)	
Secondary		1115 (28.8)	238 (28.7)	78 (41.3)	38 (48.7)	
Professional/technical training		1045 (27.0)	223 (26.9)	44 (23.3)	16 (20.5)	
University		1656 (42.8)	356 (42.9)	58 (30.7)	19 (24.4)	
Occupation N (%)						<0.001
Managerial/professional/technical		1420 (36.7)	287 (34.6)	44 (23.3)	13 (16.7)	
Clerical/services/sales		1063 (27.5)	246 (29.6)	60 (31.8)	22 (28.2)	
Skilled/unskilled manual labour		555 (14.3)	114 (13.7)	35 (18.5)	13 (16.7)	
Housework		708 (18.3)	146 (17.6)	40 (21.2)	26 (33.3)	
Student/other		127 (3.3)	37 (4.5)	10 (5.3)	4 (5.1)	
Smoking N (%)		1 (0.03)	0 (0)	0 (0)	0 (0)	0.963
Gestational weight gain (until recruitment) Mean (SD)						
Gestational weight gain (kg)		5.0 (3.0)	5.2 (2.8)	6.8 (4.4)	6.9 (4.0)	<0.001
Average weekly gestational weight gain (kg/week)		0.2 (0.1)	0.2 (0.1)	0.3 (0.2)	0.3 (0.2)	<0.001
Gestational age at birth (weeks) Median (IQR)		39 (38-40)	39 (38-40)	39 (38-40)	38 (38-40)	<0.001
	**Medical history**					
Hypertension N (%)		8 (0.2)	1 (0.1)	4 (2.1)	3 (3.9)	<0.001
	**Obstetric history**					
Parity N (%)						<0.001
Nulliparous		2133 (55.1)	426 (51.3)	69 (36.5)	31 (39.7)	
Multiparous ^a^		1740 (44.9)	404 (48.7)	120 (63.5)	47 (60.3)	
	**Among women who had previous birth/s**					
Total N (%)	2304	1735 (75.3)	403 (17.5)	120 (5.2)	46 (2.0)	
History of macrosomia N (%)		33 (1.9)	13 (3.2)	7 (5.8)	5 (10.9)	<0.001
History of preterm N (%)		198 (11.4)	50 (12.4)	11 (9.2)	7 (15.2)	0.682
History of neonatal death N (%)		97 (5.6)	27 (6.7)	6 (5)	4 (8.7)	0.680

*GDM* (diagnosis of) Gestational Diabetes Mellitus; Baseline characteristics of women with and without GDM. *P*-values used two-tailed test at the 5% significance level

^a^ Includes women who had 1-4 previous births

Obesity defined as BMI ≥ 27.5 kg/m^2^

### Maternal outcomes

Women with GDM experienced similar rates of primary C-section compared to those without GDM (35% versus 33% respectively, *p* = 0.3), Table 2. More women with GDM experienced severe pre-eclampsia, eclampsia or HELLP than women without GDM (1.3% versus 0.6%, *p* = 0.03). However, after adjustment for confounders, this difference was no longer significant (OR: 1.42, CI: 0.70–2.87).

More women with GDM delivered preterm (11% in GDM compared to 8% in non-GDM women, *p* = 0.002), and this association remained significant after adjustment for potential confounders; GDM-women were at 40% significantly increased odds of delivering preterm (OR: 1.40, CI: 1.09–1.78).

**Table 2:** Univariate and Multivariable Analysis of Maternal Outcomes by Gestational Diabetes Mellitus Status

	*Maternal Outcomes*				
*Outcome*	*Total*	*No GDM*	*GDM*	*Odds Ratio (Unadjusted)* (95% CI)	*Odds Ratio (Adjusted)* (95% CI)
*Primary C-section*	*1372*	*1117/3410 (32.8%)*	*255/738 (34.6%)*	*1.08 (0.92-1.28)*	*1.01 (0.85-1.19)*^a^
Total C-section	*2181*	*1757/4062 (43.3%)*	*424/908 (46.7%)*	1.15 (0.99-1.33)	*0.97 (0.83-1.12)*^a^
Hypertensive disorders of pregnancy	*169*	*130/4062 (3.2%)*	*39/908 (4.3%)*	*1.36 (0.94-1.96)*	1.03 (0.70-1.52)^b^
Severe pre-eclampsia, eclampsia, HELLP	*38*	*26/4062 (0.6%)*	*12/908 (1.3%)*	*2.08 (1.05-4.14)*	1.42 (0.70-2.87)^b^

*GDM* (diagnosis of) Gestational Diabetes Mellitus; Univariate and multivariable logistic regression reporting Odds Ratios with 95% confidence intervals for association of GDM with maternal outcomes.

^a^ Adjusted for: age, BMI, socioeconomic status

^b^ Adjusted for: age, BMI, gestational weight gain, socioeconomic status, parity, history of hypertension

### Neonatal outcomes

**Fig. 2:**
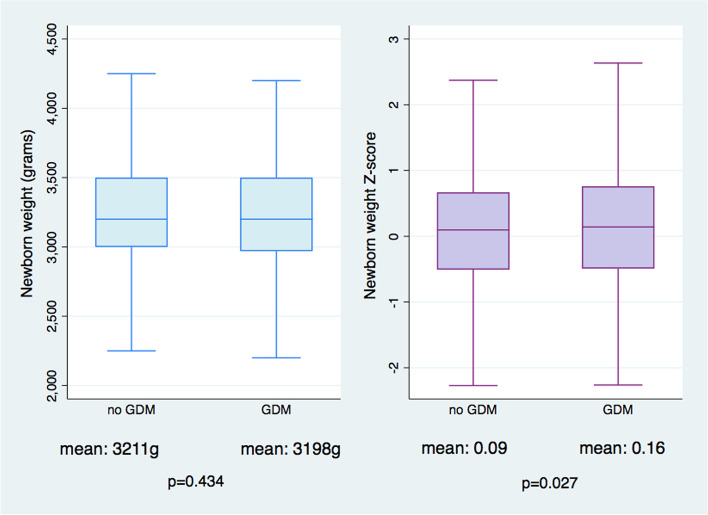
Newborn Weight by Mothers with and without Gestational Diabetes Mellitus. GDM (Gestational Diabetes Mellitus); box and whisker plot showing newborn weight (in grams) and newborn weight z-score (adjusted for gestational age at birth and newborn sex) born from mothers with and without GDM

The proportion of male babies was 52% and 50% in non-GDM and GDM-women, respectively. Whilst the mean birth weight was similar, after accounting for gestational age and gender, babies exposed to GDM had higher birthweight z-scores (0.16 compared to 0.09, *p* = 0.03), Fig. 2.

Babies exposed to GDM were also more likely born macrosomia (unadjusted OR 1.48, CI 1.05–2.08), or LGA (unadjusted OR 1.33, CI 1.05–1.69), Table 3. However, after adjustment for other confounders (maternal age, BMI, gestational weight gain, socioeconomic status, parity), a null effect could not be excluded.

**Table 3:** Univariate and Multivariable Analysis of Neonatal Outcomes by Gestational Diabetes Mellitus Status

	*Neonatal Outcomes*				
*Outcome*	*Total*	*No GDM* *N (%) or mean (SD)* ^a^	*GDM* *N (%) or mean (SD)* ^a^	*Odds Ratio or beta coefficient (Unadjusted)* *(95% CI)*	*Odds Ratio or beta coefficient (Adjusted)* *(95% CI)*
Large for gestational age	*434*	*337/4060 (8.3%)*	*97/903 (10.7%)*	*1.33 (1.05-1.69)*	1.14 (0.89-1.46)^c^
Macrosomia	*188*	*142/4060 (3.5%)*	*46/903 (5.1%)*	*1.48 (1.05-2.08)*	1.22 (0.86-1.72)^c^
Small for gestational age	*273*	*223/4060 (5.5%)*	*50/903 (5.5%)*	*1.01 (0.74-1.38)*	1.08 (0.78-1.50)^c^
Birthweight (g)		*3211 (444.19)* ^a^	*3198 (478.62)* ^a^	*-12.97* ^b^ *(-45.47 to 19.53)*	-7.81 ^b^ (-36.99 to 21.37) ^d^
Birthweight z-score		*0.087 (0.86)* ^a^	*0.158 (0.91)* ^a^	*0.071* ^b^ *(0.01 to 0.13)*	0.017 ^b^ (-0.044 to 0.079) ^c^
Preterm birth	*428*	*326/4062 (8.0%)*	*102/908 (11.2%)*	*1.45 (1.15- 1.83)*	*1.40 (1.09-1.78)* ^e^
Adverse neonatal outcome	*296*	*233/4062 (5.7%)*	*63/908 (6.9%)*	*1.23 (0.92-1.63)*	1.05 (0.78-1.42) ^f^
NICU admission	*269*	*211/4062 (5.2%)*	*58/908 (6.4%)*	*1.25 (0.92-1.68)*	*1.19 (0.87-1.61)* ^g^

*GDM* (diagnosis of) Gestational Diabetes Mellitus; Univariate and multivariable logistic and linear regression reporting Odds Ratios and beta-coefficients with 95% confidence intervals, respectively, for association of GDM with neonatal outcomes.

^a^ Mean (standard deviation)

^b^ Beta-coefficient

^c^ Adjusted for: age, BMI, gestational weight gain, socioeconomic status, parity

^d^ Adjusted for: age, BMI, gestational weight gain, socioeconomic status, parity, gestational age at birth, newborn sex

^e^ Adjusted for: age, BMI, socioeconomic status, parity, history of preterm

^f^ Adjusted for: age, BMI, socioeconomic status, gestational age at birth

^g^ Adjusted for: age BMI parity, socioeconomic status

No significant differences were observed in the individual components of neonatal mortality or severe morbidity. There was no difference in the overall composite neonatal adverse outcome (6% in non-GDM and 7% in GDM women, *p* = 0.2) or risk of NICU admission in babies born to women with GDM (5% in non-GDM and 6% in GDM women, *p* = 0.2), Table 3. Two babies suffered a fractured clavicle at birth; however, both were born to non-GDM mothers.

### Relationship between obesity, GDM and adverse outcomes

**Fig. 3:**
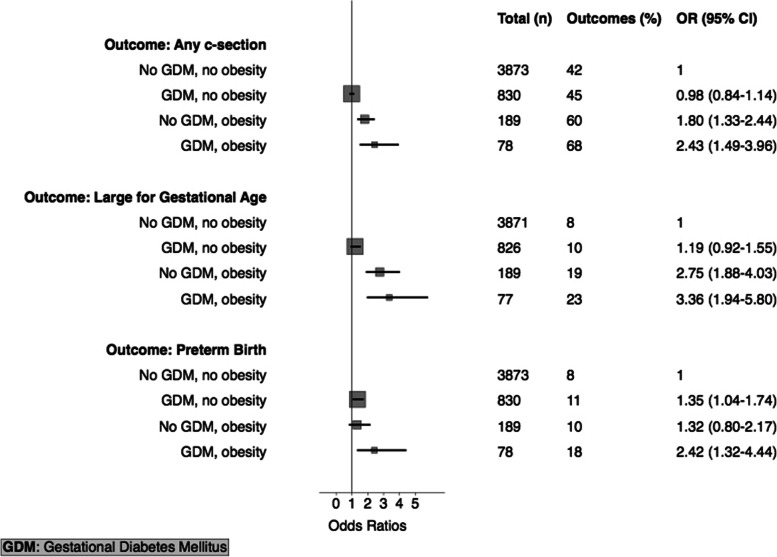
Relationship between Maternal Gestational Diabetes Mellitus, Obesity (defined: BMI ≥ 27.5 kg/m^2^), and Perinatal Outcomes. C-section adjusted for age and socioeconomic status; Large for Gestational Age adjusted for age, socioeconomic status, parity; Preterm Birth adjusted for age, socioeconomic status, parity, history of preterm birth

Overall, 267 (5.4%) women were classified as obese as per the Asian threshold, and these women were overrepresented amongst the GDM group 8.6%, compared to non-GDM 4.7%, *p* < 0.001. Compared to women without GDM or obesity, non-obese women with GDM were at increased risk of preterm birth (OR 1.35), but not C-section or LGA, Fig. 3. This contrasts with the risks observed in women who were obese without GDM. C-section and LGA were increased (OR 1.80 and 2.75), and whilst the point estimate for the risk of preterm birth was similar to in the non-obese women with GDM, a null effect cannot be excluded as the confidence interval crosses 1. The most significant risks however were observed amongst the women with both GDM and obesity. In these women, the risks of C-section, LGA and preterm birth were all increased (OR 2.43, 3.36 and 2.42, respectively).

The E-Value for GDM’s impact on preterm birth was 2.15, and for GDM and obesity’s collective impact on C-section, LGA, and preterm birth was 2.49, 3.07, and 2.49, respectively. These E-Values affirm the validity of these estimates, indicating they are relatively robust against unmeasured confounding.

## Discussion

This study aimed to elicit the burden of GDM (both singly and in combination with obesity) on adverse perinatal outcomes in an urban Asian population. As such, we used Asian-specific cut off points for BMI, as recommended by the WHO in order to more accurately reflect the synergistic adverse impact of obesity. We present evidence that women and babies affected by GDM in Ho Chi Minh City experienced higher preterm birth and were born LGA. The most striking associations however were amongst women who had GDM and were also obese, in whom the risks of C-section, LGA and preterm birth were all increased. The overall prevalence of GDM in this study was high (18%), consistent with other reports from SEA populations [9].

We add new insights into the synergistic effects of BMI, specifically obesity on perinatal outcomes, defined using an Asian specific threshold. We demonstrate that obesity, defined ≥ 27.5 kg/m^2^, and GDM act synergistically towards three pregnancy outcomes: C-section delivery, LGA, and giving birth preterm. Whilst this is consistent with the risks in obese women with GDM reported by the HAPO group [20], it is notable in our study that these risks were observed at a much lower BMI threshold (27.5 versus 33 kg/m^2^). BMI was calculated in our study at 19–22 week’s of gestation rather than before pregnancy, meaning that potentially adverse effects would have been observed at even lower pre-pregnancy BMIs. Among women who were both GDM and obese, we observed an increased risk for C-section delivery of 2.43, compared to 1.71 in the HAPO cohort, and the risk of delivering an LGA baby was 3.36 compared to 3.62 [20]. While the timing of the BMI measurements differed between our study and the HAPO cohort (19–22 weeks in this study versus 24–32 weeks gestation), we feel it is unlikely that weight gain between these gestations could account for the difference in BMI thresholds we observed (6 kg/m^2^). Our study supports the hypothesis that adverse maternal and fetal metabolic effects of GDM that are associated with obesity occur at lower BMI in Asian women.

This finding is consistent with observations outside pregnancy [21] and has implications for risk stratification in antenatal care. Asians have a higher percentage of body fat at the same BMI than their European (white) counterparts, with percentage body fat 3–5% higher than in people of European (white) ancestry for the same BMI [22, 23]. In Viet Nam, percentage body fat may be a better way to identify people at increased metabolic risk. Percentage body fat > 30 in men and > 40 in women detected around 15% of adults as obese, compared to 1.1% in men and 1.3% in women using a BMI threshold of > 30 kg/m^2^ [24]. Our findings suggest that the 30 kg/m^2^ threshold used outside pregnancy, or the 32 kg/m^2^ suggested for use in pregnancy (as per HAPO), are not sensitive indicators of pregnancy risk in this population. Given that the measurement of body fat percentage is challenging during pregnancy, we feel the lower BMI threshold is a practical alternative. This study observed that GDM mothers were at 16% increased odds for delivering LGA babies, with the association modulated by maternal obesity. Therefore, identifying the women at greatest risk of complications could help target nutritional support and intensive blood glucose monitoring in a resource constrained setting.

**Fig. 4:**
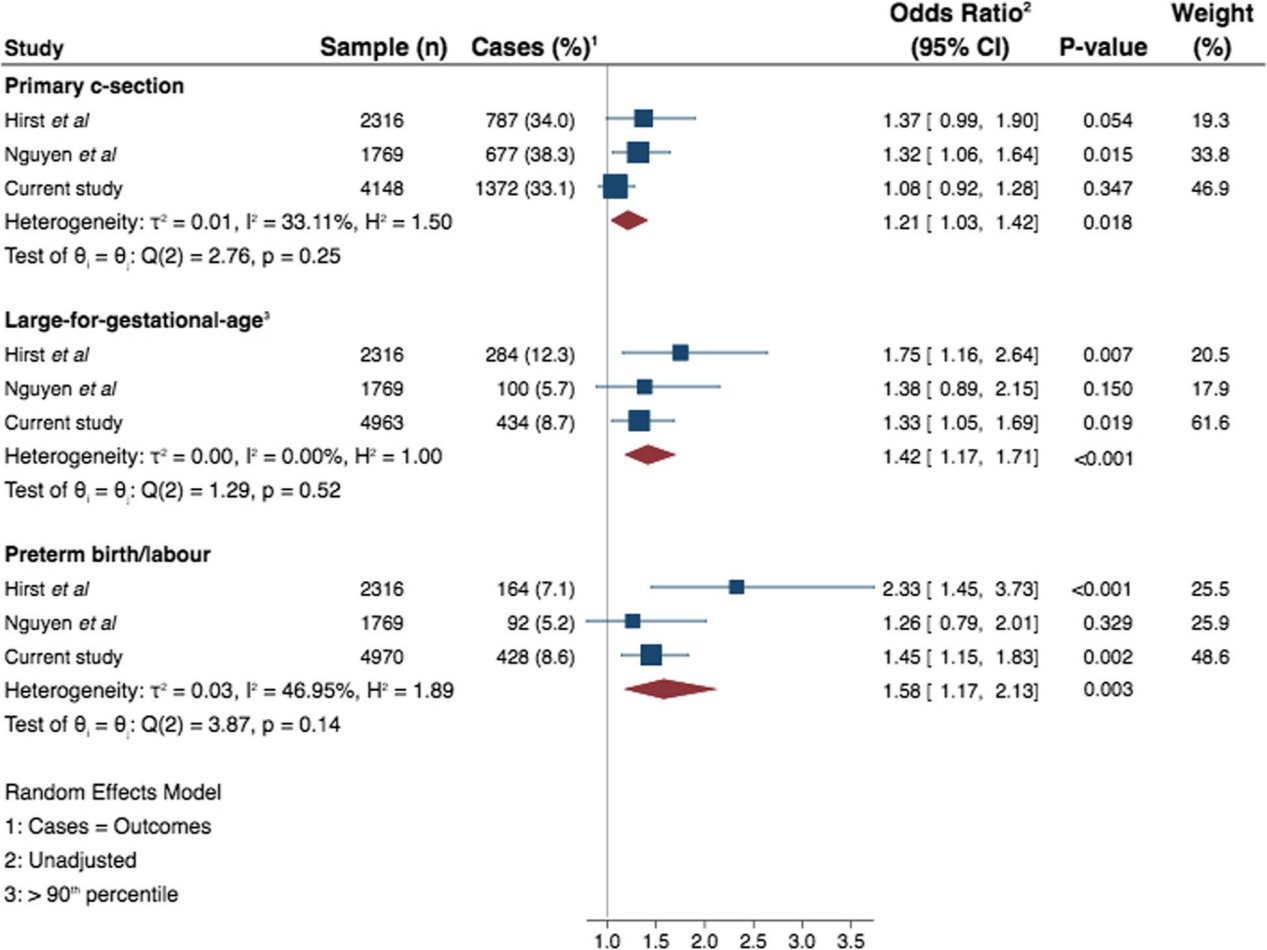
Comparison of studies examining outcomes in pregnancies affected by GDm in Viet Nam

Our work adds to a growing body of work on GDM in Viet Nam and the association with adverse perinatal outcomes (Fig. 4). Two other studies also based in urban populations reported similar findings with GDM [10, 12], although neither stratified for obesity. Associations between GDM and LGA and preterm birth have been consistently demonstrated. However, the association between GDM and C-section is less consistent. It is notable that the background C-section rate in urban Viet Nam is very high, potentially masking any effect of the GDM on likelihood of C-section birth.

The strengths of this study include a relatively large sample size, which enabled sufficient power to test for GDM’s association with primary C-section, LGA, and preterm birth. This study was conducted using a prospective design with very low loss-to-follow-up (0.2%), minimising selection bias. GDM and BMI were measured objectively, before and without knowledge of the outcomes, reducing information bias. In addition, we used international definitions of GDM (WHO/IADPSG) and size at birth (INTERGROWTH-21st ), enabling comparison with other studies. Pregnancy dating by early-pregnancy ultrasound enabled accurate estimation of risk of preterm birth and calculation of birthweight for gestational age.

We acknowledge this study has some limitations. We were unable to account for differences in individual test results of the OGTT or subsequent blood glucose monitoring and management during pregnancy. Data were also not available for neonatal hypoglycemia, an important outcome. As this was a hospital-based study, we cannot extrapolate the prevalence of GDM to the population, although the rate observed is in keeping with that from other studies in Viet Nam. Long-term effects on the mother and baby were not collected. We were not able to compare our results with previously published obesity risk definitions, as the number of women with BMI > 32 kg/m^2^ in this study was very low (*n* = 20). Whilst ideally we would have liked to compare pre-pregnancy BMI, this data was largely based on maternal recall. Since we had objective measures from recruitment (early second trimester) we chose to define BMI at this point. Our results should therefore be interpreted with caution particularly when comparing to studies where BMI was measured pre-pregnancy. While women could change BMI category between conception and 19–22 weeks, we believe that the key message of this paper that those who are obese women, and particularly obese women with GDM, are at highest risk for adverse outcomes remains valid. The relatively high reported rate of previous neonatal death (5-8.7%) was also based on maternal self-report, thus we cannot verify if there was misclassification with other forms of loss (miscarriage, stillbirth, later child death).

## Conclusions

Our findings support that BMI > 27.5 kg/m^2^measured between 19 and 22 weeks gestation can be used to risk-stratify women with GDM. The study was conducted in a resource limited setting, and thus identifying the highest risk group (i.e. women who are obese with GDM) is important for clinical prioritization. For example, obese women with GDM could be recommended more intense antenatal surveillance, earlier GDM screening, or daily blood glucose monitoring.

To our knowledge, our study is the first to specifically study the individual and synergistic effects of GDM and obesity in an Asian population using an Asian specific BMI threshold for obesity. This is important for those caring for women with GDM in SEA countries, as well as those caring for expatriates living abroad. While there may be physiological and metabolic heterogeneity among Asian populations, it is an important step to quantify perinatal risks with parameters relevant to an Asian population, as recommended by an WHO expert panel. As such, we believe our study’s implications could extend beyond women of Vietnamese ethnicity.

Before pregnancy, BMI is a potentially modifiable factor, hence public health and preventative medicine should be directed at helping women maintain their weight in the healthy range. During pregnancy, BMI risk stratification could be used to target more intensive glucose monitoring or dietary support. GDM is a complex condition, and not all women with GDM are at the same risk of adverse outcomes. Evidence is needed to understand the phenotypes of GDM in different populations. We need to move towards offering personalised care approaches in pregnancy that can also optimise the life-long health of women and their children.

## Data Availability

The datasets used and/or analysed during the current study are available from the corresponding author on reasonable request.

## Abbreviations

ACHOIS: Australian Carbohydrate Intolerance Study in Pregnant Women
BMI: Body Mass Index
CI: Confidence Intervals
CS: Caesarean section
GDM: Gestational Diabetes Mellitus
GDP: Gross Domestic Product
HAPO: Hyperglycaemia and Adverse Pregnancy Outcome
HDP: Hypertensive Disorders of Pregnancy
HELLP: Haemolysis, Elevated Liver Enzymes, and Low Platelets
IADPSG: International Association of Diabetes in Pregnancy Study Groups
LGA: Large for Gestational Age
NICU: Neonatal Intensive Care Unit
OGTT: Oral Glucose Tolerance Test
OR: Odds Ratio
PBB: Preterm Birth Biomarkers
SD: Standard Deviation
SEA: South East Asia/n
SGA: Small for Gestational Age
WHO: World Health Organisation

## References

[1] Plows JF, Stanley JL, Baker PN, Reynolds CM, Vickers MH. The Pathophysiology of Gestational Diabetes Mellitus. Int J Mol Sci. 2018;19(11). [cited 2020 Jun 9] Available from: https://www.ncbi.nlm.nih.gov/pmc/articles/PMC6274679/10.3390/ijms19113342 PMC6274679 30373146

[2] Griffith RJ, Alsweiler J, Moore AE, Brown S, Middleton P, Shepherd E, et al. Interventions to prevent women from developing gestational diabetes mellitus: an overview of Cochrane Reviews. Cochrane Database of Systematic Reviews. 2020;(6). [cited 2020 Aug 7] Available from: https://www.cochranelibrary.com/cdsr/doi/10.1002/14651858.CD012394.pub3/full?highlightAbstract=gestat%7Cdiabet%7Cdiabetes%7Cgestational 10.1002/14651858.CD012394.pub3 PMC7388385 32526091

[3] Chen I, Opiyo N, Tavender E, Mortazhejri S, Rader T, Petkovic J, et al. Non-clinical interventions for reducing unnecessary caesarean section. Cochrane Database of Systematic Reviews. 2018;(9). [cited 2020 Jul 26] Available from: https://www.cochranelibrary.com/cdsr/doi/10.1002/14651858.CD005528.pub3/full 10.1002/14651858.CD005528.pub3 PMC6513634 30264405

[4] Hartling L, Dryden DM, Guthrie A, Muise M, Vandermeer B, Donovan L (2014). Diagnostic thresholds for gestational diabetes and their impact on pregnancy outcomes: a systematic review. Diabet Med..

[5] Wendland EM, Torloni MR, Falavigna M, Trujillo J, Dode MA, Campos MA (2012). Gestational diabetes and pregnancy outcomes–a systematic review of the World Health Organization (WHO) and the International Association of Diabetes in Pregnancy Study Groups (IADPSG) diagnostic criteria. BMC Pregnancy Childbirth.

[6] Roeckner JT, Bennett S, Mitta M, Sanchez-Ramos L, Kaunitz AM. Pregnancy outcomes associated with an abnormal 50-g glucose screen during pregnancy: a systematic review and Meta-analysis. J Matern Fetal Neonatal Med. 2020;1–9. 10.1080/14767058.2019.1706473 31893960

[7] Metzger BE, Lowe LP, Dyer AR, Trimble ER, Chaovarindr U, HAPO Study Cooperative Research Group (2008). Hyperglycemia and adverse pregnancy outcomes. N Engl J Med.

[8] Hod M, Kapur A, Sacks DA, Hadar E, Agarwal M, Di Renzo GC (2015). The International Federation of Gynecology and Obstetrics (FIGO) Initiative on gestational diabetes mellitus: A pragmatic guide for diagnosis, management, and care. Int J Gynaecol Obstet.

[9] McIntyre HD, Catalano P, Zhang C, Desoye G, Mathiesen ER, Damm P (2019). Gestational diabetes mellitus. Nat Rev Dis Primers.

[10] Hirst JE, Tran TS, Do MAT, Morris JM, Jeffery HE (2012). Consequences of gestational diabetes in an urban hospital in Viet Nam: a prospective cohort study. PLoS Med.

[11] Srichumchit S, Luewan S, Tongsong T (2015). Outcomes of pregnancy with gestational diabetes mellitus. Int J Gynaecol Obstet.

[12] Nguyen CL, Lee AH, Minh Pham N, Hoang Nguyen PT, Ha AVV, Khac Chu T (2019). Prevalence and pregnancy outcomes of gestational diabetes mellitus by different international diagnostic criteria: a prospective cohort study in Vietnam. J Matern Fetal Neonatal Med.

[13] Roman AS, Rebarber A, Fox NS, Klauser CK, Istwan N, Rhea D (2011). The effect of maternal obesity on pregnancy outcomes in women with gestational diabetes. j Maternal-Fetal Neonatal Med.

[14] Barba C, Cavalli-Sforza T, Cutter J, Darnton-Hill I, Deurenberg P, Deurenberg-Yap M (2004). Appropriate body-mass index for Asian populations and its implications for policy and intervention strategies. The Lancet (British edition).

[15] Mortality rate, infant (per 1,000 live births) - Vietnam | Data [Internet]. [cited 2020 Aug 5]. Available from: https://data.worldbank.org/indicator/SP.DYN.IMRT.IN?locations=VN

[16] Bennett WL, Robinson KA, Saldanha IJ, Wilson LM, Nicholson WK (2012). High Priority Research Needs for Gestational Diabetes Mellitus. J Womens Health (Larchmt).

[17] Villar J, Ismail LC, Victora CG, Ohuma EO, Bertino E, Altman DG (2014). International standards for newborn weight, length, and head circumference by gestational age and sex: the Newborn Cross-Sectional Study of the INTERGROWTH-21st Project. Lancet.

[18] Crowther CA, Hiller JE, Moss JR, McPhee AJ, Jeffries WS, Robinson JS. Effect of Treatment of Gestational Diabetes Mellitus on Pregnancy Outcomes. New England J Med. 2005;352(24):2477–86. 10.1056/NEJMoa042973 15951574

[19] VanderWeele TJ, Ding P (2017). Sensitivity Analysis in Observational Research: Introducing the E-Value. Ann Intern Med.

[20] Catalano PM, McIntyre HD, Cruickshank JK, McCance DR, Dyer AR, Metzger BE (2012). The Hyperglycemia and Adverse Pregnancy Outcome Study. Diabetes Care..

[21] Caleyachetty R, Barber TM, Mohammed NI, Cappuccio FP, Hardy R, Mathur R (2021). Ethnicity-specific BMI cutoffs for obesity based on type 2 diabetes risk in England: a population-based cohort study. Lancet Diabetes Endocrinol.

[22] Deurenberg P, Deurenberg-Yap M, Guricci S (2002). Asians are different from Caucasians and from each other in their body mass index/body fat per cent relationship. Obes Rev..

[23] Lim U, Ernst T, Buchthal SD, Latch M, Albright CL, Wilkens LR (2011). Asian women have greater abdominal and visceral adiposity than Caucasian women with similar body mass index. Nutr Diabetes.

[24] Ho-Pham LT, Lai TQ, Nguyen MTT, Nguyen TV (2015). Relationship between Body Mass Index and Percent Body Fat in Vietnamese: Implications for the Diagnosis of Obesity. PLOS ONE.

